# Comparison of the Belgian Rheumatoid Arthritis Disability Assessment and Health Assessment Questionnaires as Tools to Predict the Need for Support Measures in Patients with Rheumatoid Arthritis

**DOI:** 10.1371/journal.pone.0146688

**Published:** 2016-01-22

**Authors:** Xavier Janssens, Saskia Decuman, Filip De Keyser, Belgian Rheumatoid Arthritis Disability Assessment study group

**Affiliations:** 1 Department of Rheumatology, Ghent University Hospital, Ghent, Belgium; 2 Department of Internal Medicine, Ghent University, Ghent, Belgium; University of Birmingham, UNITED KINGDOM

## Abstract

**Objective:**

Scores on the Health Assessment Questionnaire (HAQ) predict the need for support measures in patients with rheumatoid arthritis (RA). In this study we compare the performance of the HAQ in this context with that of the more disease-specific Belgian Rheumatoid Arthritis Disability Assessment (BRADA) questionnaire.

**Methods:**

In this multicenter observational study, patients with RA and disease duration of at least one year who consulted their rheumatologist for a routine follow-up visit filled out the HAQ, and BRADA questionnaires. The performance of HAQ and BRADA to predict the need for support measures available to patients with RA was evaluated using Receiver Operator Characteristic (ROC) curves, with the expert opinion of the rheumatologist as a reference.

**Results:**

The study analyzed data of 301 patients with RA (70.8% females) with mean age 59.8±12.8, disease duration 11.4±9.3 years, and DAS28 values of 2.84±1.18. HAQ scores averaged 0.97±0.73 and BRADA scores were 3.92±3.49 over the last week and 3.89±3.50 over the last 3 months. The area under the ROC curves for the BRADA scores for the support measures investigated ranged from 0.702 to 0.862 and did not differ significantly from those of the HAQ (range 0.725–0.860).

**Conclusion:**

The disease-specific BRADA questionnaire is equivalent to the HAQ in predicting the need for support measures in patients with stable RA.

## Introduction

Rheumatoid arthritis (RA) is characterized by chronic joint inflammation and can have a major impact on human functioning and health-related quality of life [1]. RA can lead to substantial impairment of mobility, loss of productivity and disability due to pain, fatigue and structural joint damage [2,3].

To limit the impact of the disease and safeguard patients’ quality of life, optimal care for patients with RA encompasses a combination of medical treatment targeting RA inflammatory pathways, complemented with support measures to address activity limitations, promote participation and compensate extra costs and income loss due to the disease [4,5].

Fair and equitable allocation of support measures for patients with RA warrants the need for instruments to assess the impact of the disease in a standardized way, the type and level of activity limitations and to determine the type of support measures needed in individual patients.

The Health Assessment Questionnaire (HAQ) is a well-validated general patient-reported outcome instrument developed three decades ago by Fries and colleagues at Stanford University with proven validity and reliability in patients with RA [6–10]. In the United Kingdom, the HAQ has been shown to predict successful application for benefits and financial support measures: 69% of patients with RA or osteoarthritis of hip or knee and a HAQ score >1.5 successfully applied for benefits [11–13]. In these studies, HAQ scores ≥2 [11] or ≥1.5 [12,13] predicted successful application for the Disability Living Allowance (DLA) and Attendance Allowance (AA) benefits. These are non-means tested benefits awarded to people under 65 for the DLA, and those aged 65 and over for the AA, respectively, on the basis of their need for personal care and/or their difficulties with mobility. In these studies, RA patients selected based on their HAQ scores were then assisted by a professional welfare worker in applying for the DLA or AA benefits.

We previously observed that HAQ score is a good predictor of the need for support measures in adult patients with RA and a disease duration of at least one year assessed against the expert opinion of the treating rheumatologist as a reference [14]. In this context, the HAQ performed better than SF-36 scores or DAS28 [14].

However, the HAQ is a general and not a disease-specific questionnaire. We recently developed and validated a more disease-specific questionnaire to evaluate chronic activity limitations in patients with RA, the Belgian Rheumatoid Arthritis Disability Assessment (BRADA) questionnaire [15]. In addition to the domains covered by the HAQ, the BRADA questionnaire incorporates additional elements from the International Classification of Functioning, Disability and Health (ICF) core set for RA [16–18] and assesses the level of functioning in 6 domains (mobility, nutrition, self-care, household tasks, awareness of danger and communication) over the last week and the last 3 months [15]. The BRADA questionnaire therefore represents a suitable model to challenge the performance of the HAQ in predicting the need for support measures in patients with RA.

The performance of the BRADA to assess and predict the need for support measures in patients with RA has not been investigated up to now. In this study, we therefore compare the performance of the new disease-specific questionnaire BRADA with that of the more generic HAQ in predicting the need of support measures in patients with RA. We aim to investigate whether patient questionnaire tools to assess functional limitations in patients with RA could also be used to determine the need for support measures in these patients.

## Patients and Methods

### Ethics Statement

The BRADA study was performed in different rheumatologic centers in Belgium (UZ, Gent; AZ Sint-Lucas, Gent; AZ Alma Sijsele; GHDC Hôpital Sint-Joseph Gilly, Charleroi; ULB Erasme, Brussel; UCL, Brussel; AZ Sint-Lucas, Brugge; ASZ, Aalst; GHC Saint-Joseph, Luik; UZ Gasthuisberg, Leuven; CHU Sart Tilman de Liège, Liège; Clinique Saint-Pierre, Ottignies). The ethical committee of the Ghent University Hospital acted as central EC for this study (dossier 2010/094) and approved the study on behalf of all participating centers (unique study Belgian study id covering all centers: B67020108165, date of approval: 29-Jul-2010). Approval of this EC to start the study explicitly means that all other EC of the peripheral centers have given written permission to start the study also in their center. Written informed consent was obtained from all patients before inclusion in the study.

### Study Design

The setup of this multicenter observational study was extensively described in a previous study [14]. Briefly, the study included patients of at least 18 years of age who fulfilled the American College of Rheumatology diagnostic criteria for RA [19] and had a disease duration of at least one year. No specific exclusion criteria were defined. To ensure an unbiased, representative population of patients with relatively stable RA, only the first patients in every consultation block who consulted their rheumatologist for routine outpatient evaluation were eligible for inclusion in the study. Patients who gave their written informed consent were included in the study and proceeded with the study data collection in the same visit.

### Data collection

Data collected comprised patient demographics, disease history, current treatment, DAS28 [20], short HAQ [7] and BRADA [15] questionnaires. The supplementary S1 File contains the study data and a complete list of study variables.

DAS28 is a well validated composite disease activity index for RA, calculated with the number of tender and swollen joints in a standard 28 joint count, a laboratory parameter (ESR or CRP) and optionally the patient global health VAS score as input variables. DAS28 values below 3.2 indicate low disease activity, those above 5.1 high disease activity. [21,22].

The HAQ has become a widely used patient reported outcomes tool to asses functional status in the three decades since its inception by Fries and colleagues at Stanford University, both in the context of clinical trials and in daily clinical practice [23,24]. It has been translated in many languages and has proven validity and reliability in patients with RA [9,10]. Higher scores on the HAQ indicate increasing levels of disability: scores 0 to 1 are considered to reflect mild to moderate difficulty, 1 to 2 moderate to severe disability and 2 to 3 severe to very severe disability [7].

The BRADA questionnaire is a patient reported questionnaire covering all domains of the HAQ and additionally incorporating a number of elements from the ICF core set for RA. BRADA assesses functioning in 6 domains (mobility, nutrition, self-care, household tasks, awareness of danger and communication). For each domain, the patient scores his/her ability to perform 6 activities during the last week as well as in the last three months as: without difficulty (score 0), with some difficulty (score 1), very difficult (score 2), impossible (score 3). The BRADA questionnaire yields two total scores ranging from 0 to 18 for functional status over the last week and the last three months, with higher scores indicating higher functional limitations. BRADA scores show good test-retest reliability and correlate excellently with other validated questionnaires (HAQ, SF-36) and with measures of disease activity (VAS, DAS28) [15].

The treating rheumatologists scored the need for each of 9 different supporting measures (Table 1) available for chronically ill patients in the Belgian social security system on a four item categorical scale as: certainly needed, probably needed, probably not needed or certainly not needed. HAQ and BRADA questionnaires were filled out independently by the patient. The rheumatologists were unaware of the patient’s questionnaire scores, which were only calculated during data analysis.

**Table 1:** Support measures for chronically ill patients investigated in this study.

Support measure	Type	Description
Integration allowance	Financial benefit (*<65* years)	To compensate extra costs due to diminished autonomy and to enhance social participation of person with special needs.
Allowance for help to the aged	Financial benefit (*>65* years)	For seniors with chronic health disorders confronted with supplementary costs due to loss of autonomy, in order to enhance their social participation.
Tax reduction	Tax benefit	Income tax exemption for families with one or more disabled persons.
Parking card	Promotion of mobility	For persons with mobility restrictions. Allows parking in parking places reserved for disabled persons and parking without time limit in areas of time restricted parking.
Vehicle tax waiver	Tax benefit	Exemption of car tax for disabled persons.
Free public transportation for attendant	Promotion of mobility	Free public transportation access for companions of people with mobility restrictions who can’t travel alone.
Social telephone rate	Cost reduction	Reduced telephone connection rate, subscription fee and call charges for people with functional limitations.
Social rate utility services	Cost reduction	Exemption of regular allowance and free delivery of a fixed amount of gas and electricity for disabled persons.
Allowance for chronic illness	Financial benefit (*<65* years)	Premium for persons with a chronic disease confronted with high medical costs.

### Data analysis and statistics

BRADA scores assessing functioning over the last week and the last three months were computed by averaging the six summed domain scores (mobility, nutrition, self-care, household tasks, awareness of danger and communication) resulting in two separate overall scores (covering the last week and the last three months), both with a maximum value of eighteen, with higher scores representing increasing activity limitations [15]. Certain support measures relate specifically to the activities of the mobility domain (parking card, vehicle tax waiver, free public transportation for attendant) or the communication domain (social telephone rate) of the BRADA questionnaire. Therefore, BRADA scores were additionally computed by calculating a weighted average of the summed domain scores with double weight factors for the mobility and communication domains when calculating the overall BRADA scores.

Patients were excluded from analysis when HAQ or BRADA scores could not be calculated or the rheumatologist’s recommendation on the need for support measures was missing.

Data are presented as mean±standard deviation for normally distributed variables, as median (range) for variables not following a normal distribution or as percentages. Statistical analysis was performed with SPSS version 20 software (IBM Corporation, Armonk, New York, USA). The Kolmogorov-Smirnov test was used to assess the normal distribution of variables.

The performance of the HAQ and BRADA questionnaires as tests to predict the need for support measures by patients with RA was evaluated by comparing the area under the curve (AUC) of Receiver Operator Characteristics (ROC) curves [25], with the recommendation of the treating rheumatologist for all nine support measures evaluated as the reference. Scores ‘certainly needed’ and ‘probably needed’ were considered a positive recommendation for a particular support measure. The AUCs of the ROC curves for HAQ and BRADA scores were compared using ANOVA.

A p-value <0.05 was considered statistically significant.

## Results

### Population characteristics

Informed consent was obtained from 316 patients, but the study population analyzed contained 301 patients: fifteen patients were excluded from analysis because (more than one reason possible): HAQ (n = 4) or BRADA (n = 5) scores could not be calculated or the rheumatologist’s recommendation on the need for support measures was missing (n = 8).

**Patients in this study were 59.8±12.8 years old and had an average RA disease duration of more than ten years (**Table 2).

**Table 2:** Population characteristics.

Population characteristics	Overall
Nr of patients	301
Age (y)	59.8 ± 12.8
Gender ratio (% female)	70.8
**RA characteristics **	
Disease duration (y)	11.4±9.3
RF (% positive)	73.5
Anti-CCP (% positive)	60.1
Erosions (% positive)	67.3
DAS 28	2.84 ± 1.18
**Treatment** (% of patients)	
DMARDs	86.4
DMARDs–no biologicals	50.5
Biologicals	47.2
Corticosteroids	44.2
NSAIDs	42.9

Data are expressed as mean±standard deviation or as percentages.

***Abbreviations*:** *anti-CCP*: *anti-cyclic citrullinated peptide antibody*, *DAS28*: *disease activity score using 28 joint counts*, *DMARDs*: *Disease-modifying anti-rheumatic drugs*, *NSAIDs*: *non-steroidal anti-inflammatory drugs*, *RF*: *rheumatoid factor*

The study recruited patients who consulted their rheumatologist for a routine outpatient check-up and thus consisted of patients with relatively stable disease: treatment was changed during the study visit in only 14.8% of patients and the mean DAS28 value of 2.84±1.18 indicated low disease activity.

Current RA treatment included DMARDs in most of patients (86.4%, 50.5% treated exclusively with DMARDS); biologicals were given to nearly half of the patients (47.2%).

### BRADA and HAQ scores

BRADA scores evaluating functioning over the last week averaged 3.92±3.49, whereas the BRADA scores covering the patients’ assessment of their functioning over the last 3 months were 4.12±3.51 (Table 3).

**Table 3:** BRADA and HAQ scores.

BRADA	1 week	3.92±3.49
	3 months	4.12±3.51
BRADA double weighted	1 week	3.89±3.50
	3 months	4.09±3.52
HAQ		0.97±0.73
	% HAQ >1	40.2

***Abbreviations*:** *BRADA*: *Belgian Rheumatoid Arthritis Disability Assessment*, *HAQ*: *Health Assessment Questionnaire*

BRADA double weighted scores are the results of an alternative computing of the BRADA score where the overall one week and three month BRADA scores are calculated as a weighted average of the summed domain scores, with double weight given to the mobility and communications domains. Data are expressed as mean±standard deviation or as percentages.

Additionally, BRADA total scores were computed with an alternative method as a weighted average of the domain scores giving double weight to the mobility and communications domains because four out of nine support measures investigated specifically concern activities related to the mobility or communications domain of the BRADA questionnaire. BRADA scores with double weighting for the mobility and communication domains were 3.89±3.50 for the last week and 4.09±3.52 for the last 3 months, respectively.

HAQ scores were 0.97±0.73 and 40.2% of patients (n = 121) had a HAQ score >1 (Table 3).

### Comparison of BRADA and HAQ as predictors for the need of support measures

**Fig 1:**
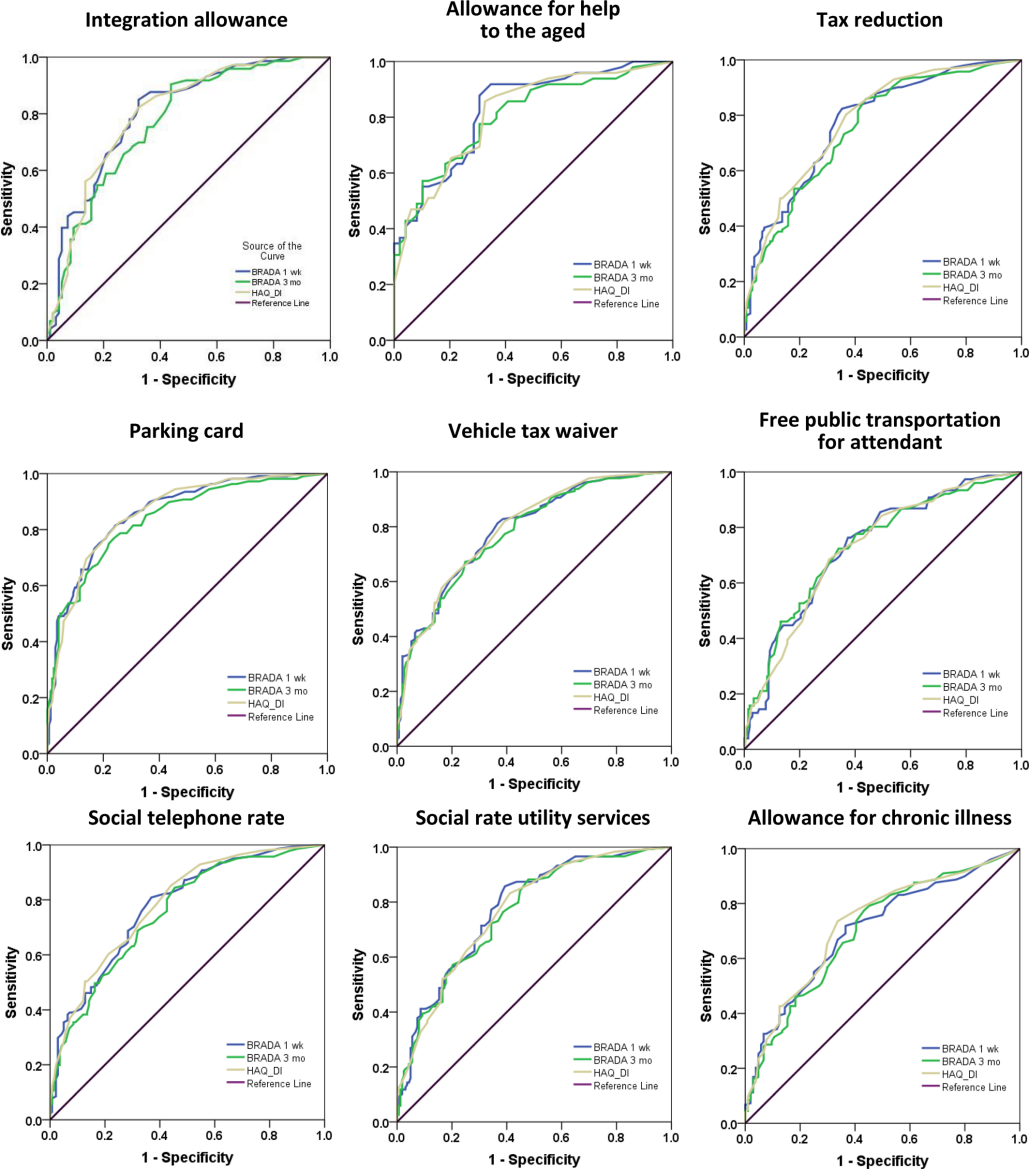
ROC curves comparing BRADA and HAQ as predictors for the need of support measures in patients with RA. Rheumatologists evaluated their patients’ need for each of the nine support measures currently available in Belgium. Receiver Operator Characteristics Curves (ROC) comparing the performance of the BRADA questionnaire scores with the HAQ in predicting the need for support measures, taking the positive recommendation of the rheumatologist as a reference. These ROCs show that BRADA and HAQ perform comparably well for all support measures evaluated.

**ROC curves for every support measure investigated are presented in** Fig 1. The AUCs of these ROC curves are summarized in Table 4. The ROC curve AUCs were consistently >0.7 for all support measures investigated (range 0.702–0.862 for BRADA and 0.725–0.860 for HAQ) and did not differ significantly for BRADA and HAQ (ANOVA, p = 0.787).

**Table 4:** Area under the curve of Receiver operator curves comparing the performance of BRADA and HAQ scores in predicting the need for support measures in patients with RA.

	BRADA		BRADA—Double Weighted		HAQ
	1 week	3 months	1 week	3 months	
Integration allowance (<65 y)	0.806	0.771	0.805	0.773	0.801
	(0.740–0.871)	(0.701–0.841)	(0.739–0.871)	(0.703–0.843)	(0.735–0.876)
Allowance for help to the aged (>65 y)	0.829	0.805	0.836	0.809	0.815
	(0.749–0.909)	(0.720–0.891)	(0.758–0.914)	(0.724–0.894)	(0.732–0.899)
Tax reduction	0.777	0.755	0.776	0.754	0.783
	(0.723–0.831)	(0.699–0.811)	(0.722–0.830)	(0.698–0.810)	(0.730–0.836)
Parking card	0.862	0.840	0.866	0.845	0.860
	(0.818–0.905)	(0.792–0.888)	(0.814–0.909)	(0.798–0.891)	(0.816–0.903)
Vehicle tax waiver	0.790	0.779	0.795	0.787	0.793
	(0.738–0.842)	(0.726–0.833)	(0.743–0.846)	(0.734–0.839)	(0.741–0.844)
Free public transportation for attendant	0.734	0.733	0.741	0.738	0.727
	(0.669–0.798)	(0.667–0.799)	(0.677–0.804)	(0.673–0.804)	(0.662–0.791)
Social telephone rate	0.776	0.754	0.773	0.751	0.785
	(0.723–0.830)	(0.698–0.810)	(0.720–0.827)	(0.695–0.807)	(0.732–0.837)
Social rate utility services	0.776	0.76	0.779	0.761	0.769
	(0.723–0.830)	(0.705–0.816)	(0.726–0.833)	(0.705–0.816)	(0.714–0.823)
Allowance for chronic illness	0.706	0.702	0.707	0.701	0.725
	(0.645–0.767)	(0.639–0.765)	(0.645–0.768)	(0.639–0.763)	(0.664–0.786)

AUCs of ROC curves (95% confidence interval) for the BRADA and HAQ questionnaires were comparable (ANOVA, p = 0.787). The recommendation of the treating rheumatologist for each support measure analyzed was used as a reference.

ROC curves comparing BRADA and HAQ in predicting the need for support measures were also constructed using the BRADA scores calculated as weighted average with double weighting for the mobility and communications domains (data not shown). This alternative computation of the BRADA scores yielded results comparable to those of the standard BRADA computation: AUCs of the ROC curves for both computation methods did not differ significantly. Neither did the AUCs for BRADA scores with double weighting of the mobility and communications domains differ significantly from those of the HAQ (Table 4).

## Discussion

Patients with RA are confronted with activity limitations, participation issues and additional costs [4,26–28]. Optimal care for these patients therefore needs to encompass both medical treatment and support measures to compensate the difficulties they experience in performing daily activities and participating in society.

In this study we compared the performance of the disease-specific BRADA questionnaire with the well-known and widely used generic HAQ for predicting the need for support measures in patients with stable, longstanding RA.

The BRADA questionnaire was found to be a suitable tool for predicting the need for support measures in patients with RA as assessed by their rheumatologist, as ROC curve AUCs were consistently above 0.7 for all support measures investigated. Somewhat surprisingly, performance of the BRADA questionnaire in this context was equivalent to but not better than that of the HAQ.

The BRADA questionnaire encompasses all eight domains of the HAQ and additionally contains a number of elements from the ICF core set for RA, that were added to make the questionnaire disease-specific. The additional ICF elements added mainly involve mobility (such as riding a bicycle, driving a car, moving around the house), self-reliance (e.g. get up after a fall, get medication out of the package and take it) and communication (e.g. use a computer, use a telephone) or participation items such as the ability to go to public buildings or participate in a one-day outing.

Possibly, the incorporated ICF elements did not significantly change the performance of the BRADA questionnaire to predict the need of certain support measures in this study over that of the HAQ since the study population comprised stable patients with relatively low disease activity (DAS28 of 2.84 ± 1.18). It is as yet unclear whether the results of this study can be extrapolated to a population of RA patients with higher disease activity.

The fact that the BRADA is designed to estimate functioning over the last week as well as over the last three months targets the scope of this questionnaire toward the evaluation of stable long-term activity limitations and disability in patients with RA. The low disease activity and relatively low HAQ scores in the study population indicate that most patients with RA, even those with long-standing disease, function well under treatment.

The current study does not include data on actual support applications or allocations. If available, such data could have been used as reference in an additional ROC curve analysis to investigate the performance of the BRADA and HAQ questionnaires in predicting (successful) application for support measures. However, such analysis would compare these questionnaires to existing application and allocation procedures, which may not always be adequately tailored to the needs of patients with rheumatoid arthritis.

As patient reported outcomes measures, both HAQ and BRADA questionnaires inherently carry an element of subjectivity. However, patient questionnaire scores for physical function were shown to be superior to laboratory tests and radiographs in predicting severe outcomes in RA such as mortality and work disability [29].

Although further research is needed in order to investigate optimal cut-off values in populations with different disease severity, and to determine which level of questionnaire scores optimally predicts the need for specific support measures, the current study demonstrates that patient questionnaires HAQ and BRADA are reliable, standardized tools to measure activity limitation in patients with RA, both as clinical follow up instruments and as tools to assess the need of support measures.

The main impact and relevance this study and research on the use of patient questionnaires to predict the need of additional support measures in patients with RA may have on clinical practice in rheumatology, is to raise rheumatologists’ awareness. Awareness that first and foremost patients with RA are in need of support–in additon to optimal medical treatment and often even when remission has been achieved—to alleviate the consequences of the disease on their quality of life. Awareness that they as rheumatologists are in a good position to inform their patients and direct them to additional support measures, using the same instruments (questionnaires such as BRADA and HAQ) they use to assess activity limitations of their patients.

Further research is needed to confirm our findings in populations in populations with RA with different disease activity and disability levels, and establish suitable threshold scores for different types of support measures; Rasch analysis [30] of the BRADA questionnaire will be needed to identify the optimal combination of items to investigate functioning in populations with different levels of functional impairment or disability.

This type of research ultimately may have an impact on the policies government support agencies use to allocate support measures to patients and to distribute the funds for these support measures equitably and according to objective criteria to those most in need of these support measures.

In summary, disease-specific BRADA questionnaire is able to adequately predict the need for support measures in patients with stable RA and performs equally well but not better than the HAQ in this context.

## Supporting Information

S1 File Complete Study Data. Data file containing all reported parameters for all patients included in the study. Variable names, descriptions and datatypes are inlcuded on a separate sheet.
(XLSX)
